# Zero-fluoroscopy pulsed field ablation of atrial fibrillation in a patient with cor triatriatum sinister: A case report

**DOI:** 10.1016/j.hrcr.2026.01.002

**Published:** 2026-01-10

**Authors:** Yosuke Mizuno, Yoshiko Munehisa, Daiki Kumazawa, Kosuke Onodera, Kazuhiro Satomi, Kennosuke Yamashita

**Affiliations:** 1 Heart Rhythm Center, Department of Cardiovascular Medicine, Sendai Kousei Hospital, Miyagi, Japan; 2 Department of Cardiovascular Medicine, Sendai Kousei Hospital, Miyagi, Japan; 3 Department of Cardiology, Tokyo Medical University, Tokyo, Japan

**Keywords:** Catheter ablation, Atrial fibrillation, Pulsed field ablation, Cor triatriatum sinister, 3-Dimensional electroanatomic mapping, Zero fluoroscopy

**Key Teaching Points**

▪ Pulsed field ablation provides a safe and effective strategy for pulmonary vein isolation in cor triatriatum sinister owing to its myocardial selectivity and low risk of collateral injury.
▪ Real-time intracardiac echocardiography is essential for understanding the left atrial membrane and ensuring safe transseptal puncture in patients with cor triatriatum sinister.
▪ Even without fluoroscopy, the combination of intracardiac echocardiography and 3-dimensional electroanatomic mapping allows pulsed field ablation to be performed safely in patients with complex atrial structures.

## Introduction

Cor triatriatum sinister (CTS) is a rare congenital cardiac anomaly in which a fibromuscular membrane divides the left atrium (LA) into posterosuperior and anteroinferior compartments, typically communicating through 1 or more fenestrations.^1^ The membrane often remains undetected during childhood and may first be identified in adulthood, where it can present with atrial fibrillation (AF), heart failure, or other atrial pathology owing to hemodynamic features resembling mitral stenosis. In patients from midlife to older age whose only indication for intervention is atrial arrhythmia, catheter ablation has been described in isolated case reports.^2^, ^3^, ^4^ CTS presents unique technical challenges for AF ablation: transseptal puncture can be difficult, and catheter manipulation within the LA is often restricted by the membrane and its fenestrations. To the best of our knowledge, there have been no previous reports of pulsed field ablation (PFA) for a patient with CTS. We report a rare case of paroxysmal AF with CTS successfully treated with zero-fluoroscopy PFA using the PulseSelect system (Medtronic).

## Case report

**Figure 1:**
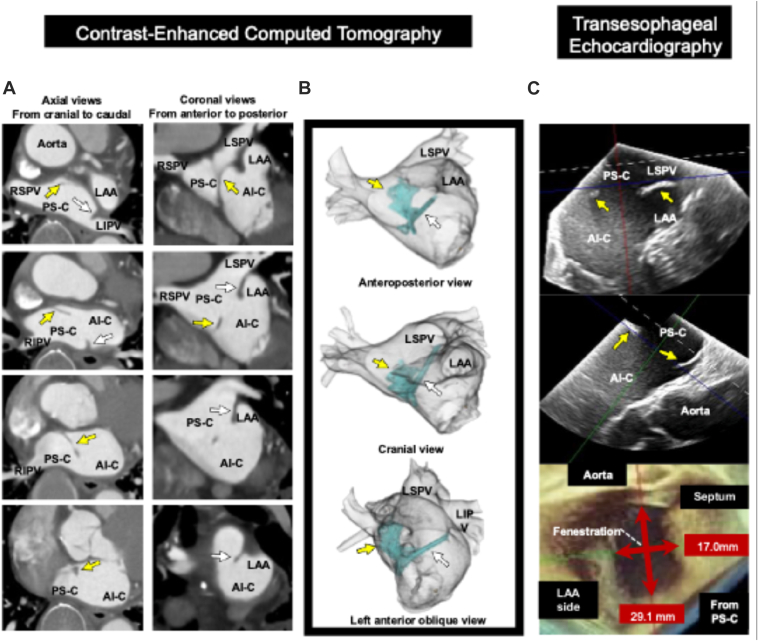
Preablation imaging of the LA membrane and fenestration. **A:** Contrast-enhanced computed tomography showing the LA membrane. **B:** 3-dimensional reconstruction of contrast-enhanced computed tomography demonstrating the LA membrane. **C:** Transesophageal echocardiography identifying the LA membrane and fenestration (17.0 × 29.1 mm). *Yellow and white arrows*: LA membrane. AI-C = anteroinferior compartment; LA = left atrium; LAA = left atrial appendage; LIPV = left inferior pulmonary vein; LSPV = left superior pulmonary vein; PS-C = posterosuperior compartment; RIPV = right inferior pulmonary vein; RSPV = right superior pulmonary vein.

A 77-year-old man with hypertension and symptomatic paroxysmal AF and atrial flutter underwent preprocedural contrast-enhanced computed tomography (CECT) for initial catheter ablation, which revealed a linear structure in the LA extending from the ridge between the left pulmonary vein (PV) and the LA appendage (LAA) toward the lower anterior interatrial septum, with a membranous extension toward the roof of the LA (Figure 1A and 1B). No other structural heart disease or vascular anomalies were identified. Transthoracic echocardiography could not visualize this structure; the LA diameter was 40 mm, the left ventricular ejection fraction was 68%, and only mild valvular disease was present.

Transesophageal echocardiography (TEE) revealed a membranous structure extending from the left superior PV–LAA ridge to the anterior margin of the inferior fossa ovalis, with a distinct fenestration in its central-to-lateral upper portion measuring 29.1 × 17.0 mm and 4.07 cm^2^ (Figure 1C). The membrane divided the LA into a posterosuperior compartment, containing the posterior wall and PV orifices, and an anteroinferior compartment, containing the LAA and mitral annulus, consistent with CTS. 3-dimensional CECT showed the membrane as a linear border, unlike TEE, likely because some parts were too thin for visualization (Figure 1B and 1C). Based on both the TEE and CECT findings, a transseptal approach for PV isolation (PVI) was determined to be feasible, and PFA was planned for paroxysmal AF.

**Figure 2:**
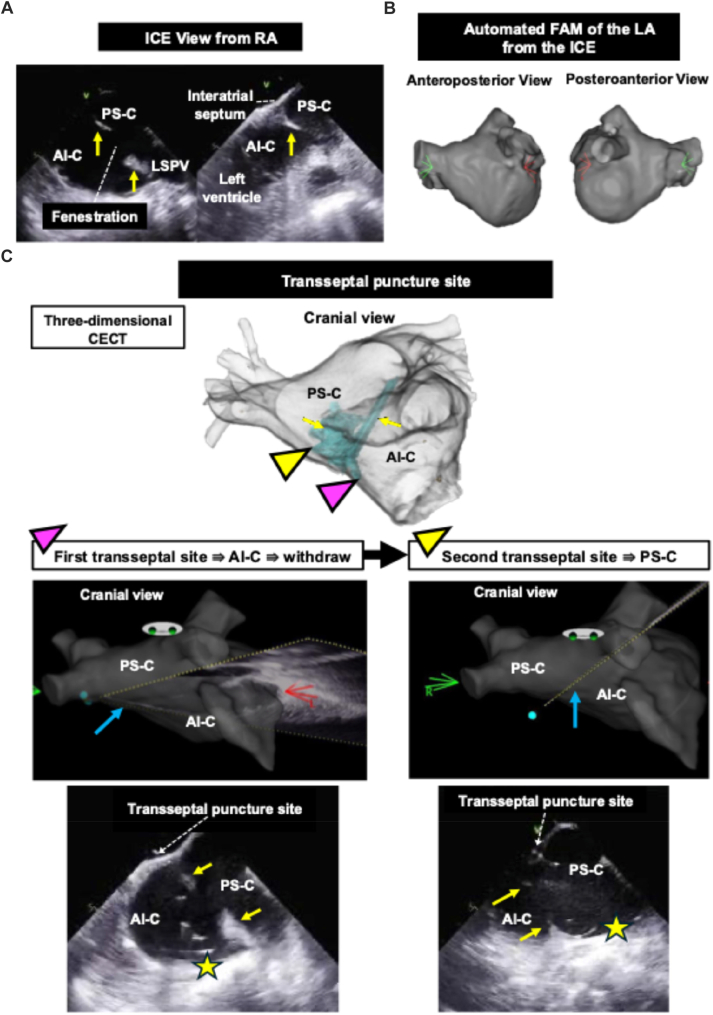
ICE and 3-dimensional imaging of cor triatriatum sinister. **A:** ICE from the right atrium showing the left atrial membrane attached to the anteroinferior fossa ovalis, forming a fenestration between the AI-C and the PS-C. **B:** Automated FAM of the LA generated from ICE. **C:** Transseptal puncture guided by ICE and 3-dimensional computed tomography. The first puncture entered the AI-C, whereas the second, targeted above the membrane, successfully accessed the PS-C. *Yellow arrows*: the LA membrane. *Yellow star*: Versacross radiofrequency wire. *Blue arrows*: the direction and position of the ICE imaging beam in the 3-dimensional mapping system. *Pink triangle*: first transseptal puncture site. *Yellow triangle*: second transseptal puncture site. AI-C = anteroinferior compartment; CECT = contrast-enhanced computed tomography; FAM = fast anatomic map; ICE = intracardiac echocardiography; LA = left atrium; LSPV = left superior pulmonary vein; PS-C = posterosuperior compartment; RA = right atrium.

The catheter ablation was performed under general anesthesia with 3-dimensional electroanatomic mapping (3D-EAM) (CARTO 3; Biosense Webster). 2 right femoral venous accesses were obtained: one for intracardiac echocardiography (ICE) (SoundStar; Biosense Webster) using a 10F, 30-cm long sheath and the other for transseptal access using an 8.5F Versacross transseptal sheath (Boston Scientific, Marlborough, MA). During the procedure, intravenous heparin maintained an activated clotting time of >300 seconds. With SoundStar, the LA membrane was visible from the right atrium, with its septal edge attached to the anteroinferior aspect of the fossa ovalis (Figure 2A). An automated fast anatomic map (FAM) of the LA was then generated using the CARTOSOUND FAM module (Biosense Webster) from the right atrium and right ventricular outflow tract, which reproduced the overall anatomy comparable with CECT, but the membrane was excluded from the reconstruction (Figure 2B).

Initially, a Versacross radiofrequency wire (Boston Scientific) was advanced under ICE guidance, targeting just above the membrane attachment on the inferior fossa ovalis for transseptal puncture. However, once the wire entered the LA, it was found by ICE to have advanced into the anteroinferior compartment (Figure 2C). Because this would limit PV access, we withdrew the wire and performed a repeat transseptal puncture, targeting the central portion, not the inferior portion, of the fossa ovalis just above the membrane attachment, allowing access to the posterosuperior compartment and smooth delivery of the sheath. The ICE beam orientation in the 3D-EAM also confirmed that the second transseptal puncture was directed posteriorly compared with the first (Figure 2C).

**Figure 3:**
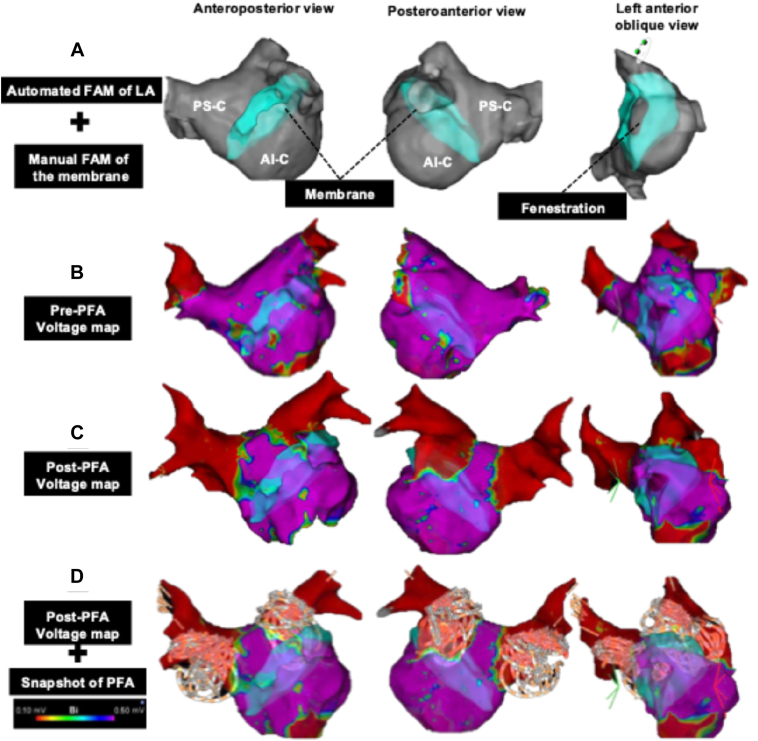
3-dimensional electroanatomic maps of the LA using the CARTO 3 system. **A:** Automated FAM of the LA merged with the manually created FAM of the membrane, demonstrating the PS-C, AI-C, and fenestration. **B:** Pre-PFA voltage maps during sinus rhythm. **C:** Post-PFA voltage maps showing complete pulmonary vein isolation. **D:** Post-PFA voltage maps overlaid with snapshots of each PFA application, showing concordance between application sites and lesion sets. AI-C = anteroinferior compartment; FAM = fast anatomic map; LA = left atrium; PFA = pulsed field ablation; PS-C = posterosuperior compartment.

A manual FAM of the membrane was created using the SoundStar and merged with the automated FAM (Figure 3A). A fenestration was identified, measuring 21.8 × 10.5 mm (2.1 cm^2^), which was smaller than that measured by TEE, possibly owing to differences in imaging resolution and perspective between modalities.

A multielectrode mapping catheter (Octaray 3-3-3; Biosense Webster) was used to obtain magnetic field data and create a voltage map during sinus rhythm of the LA (Figure 3B). Mapping of the anteroinferior compartment was performed by advancing the catheter through the fenestration under ICE guidance (Supplemental Figure 1), whereas mapping of the posterosuperior compartment was performed without difficulty. No large low-voltage areas (<0.5 mV) were found in the LA, and no electrical activity was recorded on the membrane. In addition, an impedance map obtained with a contact force–sensing catheter (QDOT MICRO; Biosense Webster), which was also used for cavotricuspid isthmus ablation, was created to assess the structural and electrophysiological properties of the membrane and showed no impedance change upon membrane contact (Supplemental Figure 2). After integrating these findings, the membrane was considered to consist of fibrous rather than myocardial tissue.

After ICE visualization of the LA, the transseptal sheath was exchanged for a bidirectional deflectable sheath (FlexCath Contour; Medtronic), and the circle array type PFA catheter (PulseSelect) was introduced into the LA over a 0.032-inch guidewire (GW1142; Medikit). Although the PulseSelect system is designed for fluoroscopic guidance, at our center, the catheter and guidewire are registered and displayed on 3D-EAM, enabling a zero-fluoroscopy workflow (Supplemental Figure 3).^5^ Preoperatively, the PulseSelect catheter was registered on the CARTO 3 system as having a 9-electrode circular design, 25-mm diameter, 3.75-mm interelectrode spacing, 3-mm electrode width, and 5F catheter size. The guidewire was also registered with 2 electrodes, 5-mm tip spacing, 1.0-mm electrode width, and 2F catheter size. This visualization, combined with ICE imaging, permitted safe navigation within the LA without fluoroscopy.

Adequate catheter–tissue contact was assessed by 2 methods: visualization with ICE and confirmation on 3D-EAM that the center electrode, designed with a forward tilt, deflected backward when pressed against the tissue and remained stable without further movement. Using 3D-EAM, the guidewire was advanced into PV, and PFA applications were delivered at the ostium and antrum of each PV during sinus rhythm. Snapshots of the catheter position were recorded after each application to document the lesion set. PFA was delivered as 11 applications in the left superior PV, 10 in the LIPV, 12 in the right superior PV, and 8 in the right inferior PV, totaling 41 applications. All PVs were accessed from the posterosuperior compartment without difficulty, and no issues occurred with guidewire advancement or PulseSelect manipulation, including to the right inferior PV via the over-the-wire system. Postablation mapping with the Octaray catheter confirmed electrical isolation of all PVs with both entrance and exit block, and absence of unexpected lesions outside the snapshots of each PFA application (Figure 3C and 3D). Subsequently, cavotricuspid isthmus ablation was performed using the QDOT MICRO catheter, achieving bidirectional block. The total time required for PVI was 23 minutes, and the total procedure duration was 146 minutes. The procedure was completed entirely without fluoroscopy or complications.

The patient was discharged the next day in sinus rhythm. At a 3-month follow-up, no recurrence of atrial arrhythmias or complications was observed.

## Discussion

To the best of our knowledge, this case represents the first report of zero-fluoroscopy PFA for CTS. PFA is a novel ablation modality that achieves myocardial-selective ablation through irreversible electroporation and has been shown to be noninferior to conventional thermal ablation in terms of both safety and efficacy.^6^ Similar to previously reported cases using conventional thermal ablation for CTS, we were able to complete PVI without procedural complications.^2^, ^3^, ^4^ Moreover, because PFA carries a very low risk of injury to surrounding structures such as the esophagus and phrenic nerve, it reduces specific intraprocedural concerns during PVI, which may be considered an important advantage in this complex anatomy. We have also reported successful PFA in cases with interrupted inferior vena cava via superior approach and with a common inferior PV, both without complications.^7^^,^^8^ However, there are no large series describing ablation in CTS, and the long-term efficacy and safety in this patient population remain unknown, warranting further investigation.

Preprocedural recognition of CTS is important, as shown by the membrane being identified on CECT in this case; however, such findings may be missed in patients with renal dysfunction or limited imaging. Therefore, intraprocedural real-time ICE views are essential to ensure accurate diagnosis and a safe ablation strategy.

The CARTOSOUND FAM module with a SoundStar uses deep learning to provide rapid, automated FAM of the LA without manual contouring, and its accuracy has been shown to be comparable with CECT.^9^ Although automated FAM delineated the LA well, the membrane was not integrated, highlighting the importance of real-time ICE. During the first transseptal puncture, although ICE guidance was used, the puncture was directed toward the inferior portion of the fossa ovalis above the membrane to facilitate access to the right inferior PV, which inadvertently resulted in entry into the anteroinferior compartment—a finding not apparent without ICE. Detection before sheath insertion prevented complications; however, PVI from the anteroinferior compartment would be difficult, underscoring the critical role of ICE in guiding safe and accurate access. Meanwhile, the anatomic patterns of CTS vary widely, and transseptal puncture may pose no problem in some cases; however, when PVI is intended, direct access to the posterosuperior compartment is generally preferable. Nonetheless, both preprocedural imaging and intraprocedural ICE evaluation are crucial for safe and effective intervention.

At our institution, ablation procedures—including PFA—are routinely performed with minimized or zero fluoroscopy using ICE and 3D-EAM, even in complex anatomies.^5^^,^^7^^,^^8^ Zero-fluoroscopy PFA using the PulseSelect system has already been reported.^10^ However, this report demonstrates that, even in a complex anatomy such as CTS, a zero-fluoroscopy procedure can be safely and effectively performed by combining 3D-EAM and ICE guidance. The zero-fluoroscopy approach eliminates radiation exposure to both patients and staff, obviates the need for lead apron use, and reduces physical strain on operators. To the best of our knowledge, all previously reported interventions for CTS—such as LAA occlusion or non-AF ablations—have required fluoroscopy, with no previous reports using low or zero-fluoroscopy approaches. Moreover, in CTS, anatomic assessment is challenging with fluoroscopy alone and typically requires LA angiography, which carries risks of embolic events and renal dysfunction.^4^ By combining 3D-EAM with ICE, detailed anatomic visualization of the LA is achievable without fluoroscopy, potentially reducing patient burden. Limitations of this workflow include the need for previous experience with fluoroscopy-guided ablation, making it less suitable for beginners, and the requirement for magnetic field acquisition to visualize devices such as the PulseSelect catheter and guidewire, which lack magnetic sensors.

When minimizing fluoroscopy, careful attention to the guidewire position and related aspects is essential for procedural safety. In our previous near-zero-fluoroscopy PulseSelect PFA series, no complications occurred.^5^ Although this case was performed with complete zero fluoroscopy, fluoroscopy should be promptly used if any resistance or abnormal device behavior is encountered to ensure safety.

## Conclusion

Zero-fluoroscopy PFA using the PulseSelect system was safely performed for AF in a patient with CTS, guided by 3D-EAM with ICE, without complications.

## Disclosures

The authors have no conflicts of interest to disclose.

## Footnotes

Appendix Supplementary data associated with this article can be found in the online version at https://doi.org/10.1016/j.hrcr.2026.01.002.
