# Supplementary material for: Zero-fluoroscopy pulsed field ablation of atrial fibrillation in a patient with cor triatriatum sinister: A case report
Source: HeartRhythm Case Rep. 2026 Jan 10;12(4):426–31. doi: 10.1016/j.hrcr.2026.01.002 (PMC13100610; doi:10.1016/j.hrcr.2026.01.002)
Supplement: Supplemental Figure Legends [file mmc1.docx]

**Supplemental Figure 1:** (A) Intracardiac echocardiography showing the Octaray^TM^ catheter positioned in the antero-inferior compartment through the fenestration. (B) Three-dimensional mapping demonstrating the Octaray^TM^ catheter in the antero-inferior compartment, consistent with the approach through the fenestration seen on echocardiography.

**Supplemental Figure 2:** Ablation catheter in the left atrium. (Left) Tip positioned in the blood pool. (Right) Tip in contact with the membrane, showing no impedance change

**Supplemental Figure 3:** CARTO^TM^ 3 registration of the PulseSelect^TM^ catheter and guidewire for three-dimensional mapping display
